# Systematic Detection of Epistatic Interactions Based on Allele Pair Frequencies

**DOI:** 10.1371/journal.pgen.1002463

**Published:** 2012-02-09

**Authors:** Marit Ackermann, Andreas Beyer

**Affiliations:** Cellular Networks and Systems Biology, Biotechnology Center, Technische Universität Dresden, Dresden, Germany; The Wellcome Trust Centre for Human Genetics, University of Oxford, United Kingdom

## Abstract

Epistatic genetic interactions are key for understanding the genetic contribution to complex traits. Epistasis is always defined with respect to some trait such as growth rate or fitness. Whereas most existing epistasis screens explicitly test for a trait, it is also possible to implicitly test for fitness traits by searching for the over- or under-representation of allele pairs in a given population. Such analysis of imbalanced allele pair frequencies of distant loci has not been exploited yet on a genome-wide scale, mostly due to statistical difficulties such as the multiple testing problem. We propose a new approach called Imbalanced Allele Pair frequencies (ImAP) for inferring epistatic interactions that is exclusively based on DNA sequence information. Our approach is based on genome-wide SNP data sampled from a population with known family structure. We make use of genotype information of parent-child trios and inspect 3×3 contingency tables for detecting pairs of alleles from different genomic positions that are over- or under-represented in the population. We also developed a simulation setup which mimics the pedigree structure by simultaneously assuming independence of the markers. When applied to mouse SNP data, our method detected 168 imbalanced allele pairs, which is substantially more than in simulations assuming no interactions. We could validate a significant number of the interactions with external data, and we found that interacting loci are enriched for genes involved in developmental processes.

## Author Summary

Elucidating non-additive (epistatic) interactions between genes is crucial for understanding the molecular mechanisms of complex diseases. Even though high-throughput, systematic testing of genetic interactions is possible in simple model organisms, such screens have so far not been successful in mammals. Here, we propose a computational screening method that only requires genotype information of family trios for predicting genetic interactions. We tested our framework on a set of more than 2,000 heterozygous mice and found 168 imbalanced allele pairs, which is substantially more than expected by chance. We confirmed many of these interactions using data from recombinant inbred lines. The number of significant allele pair imbalances that we detected is surprisingly large and was not expected based on the published evidence. Our framework sets the stage for similar work in human trios.

## Introduction

The simultaneous perturbation of two epistatically interacting genes leads to a phenotype that is not expected based on the phenotypes of the individual genes. Understanding these phenomena is indispensable for explaining multi-factorial traits and diseases [1]. In addition, epistatic interactions provide important insights into the functional organization of molecular pathways [2], [3]. Much effort has therefore been put into the development of methods to discover epistatic interactions, mostly in linkage and association studies [1], [4]–[10].

Epistasis is always defined with respect to a specific phenotype and describes a non-additive interaction effect of two genes on that phenotype. Most gene interaction studies explicitly measure a phenotype such as growth rate or viability [11]–[14]. However, one can also study implicit phenotypes by searching for the over- or under-representation of certain allele pairs in a given population. Such allele pairs are examples of Dobzhansky-Müller incompatibilities: they establish a fitness bias in favor of individuals inheriting the over-represented allele combination [15]. In their most extreme form such incompatibilities are embryonic lethal. Genes harboring these alleles are clearly in epistasis, as none of the alleles alone has a fitness effect. Only the presence of specific allele pairs in one individual exposes the phenotype. In this context, an implicit phenotype is a trait that is not explicitly measured in the sample but whose regulators can still be inferred from the genotype data.

Whereas several such incompatibilities are known in plants (see [16] and references therein), only very few allele incompatibilities have been reported in mammals [17], [18]. A small number of recent studies have explored this idea for the genome-level identification of epistatic interactions: if a large number of individuals is genotyped at a large number of genomic positions, it becomes possible to test all allele pairs for over- and under-representation in that population [18]–[20]. For example, [19] provide a map of distant linkage disequilibrium (LD) in mouse recombinant inbred lines (RIL) giving some indication about the distribution of imbalanced allele pair frequencies in the genome. However, even though some methodological progress has been made [18], previous studies could hardly identify a significant number of interactions. The main obstacle is the humongous number of statistical hypotheses tested when comparing all markers in a genome against all markers. When correcting for multiple hypothesis testing one is usually left with very few or even no significant allele pairs.

Here, we propose to address this problem by exploiting the additional information gained from studying family trios. We show that by analyzing a sufficiently large number of individuals with known family structure it becomes possible to detect substantially more interactions than what is expected if all markers were independent.

Our method, called “Imbalanced Allele Pair frequencies (ImAP)”, relies on sequence data only, making it applicable to the many already available SNP studies without the need for additional phenotype measurements. ImAP is based on inspecting 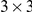 contingency tables that track the frequencies of all possible two-locus allele combinations in heterozygous individuals (assuming a diploid genome). The test that we propose is similar to a 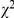 test in that it compares the observed frequencies in this table to expected frequencies assuming independence. However, our version corrects the expected frequencies for confounding factors such as family structure or allelic drift [21].

In a population of 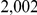 heterozygous mice with known family structure genotyped at 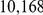 markers we identify 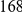 LD block pairs with imbalanced alleles. Using simulations we can show that this number is significantly larger than expected under the null hypothesis even after correcting for multiple hypothesis testing. The significance of the top scoring interactions between the LD blocks could be independently confirmed using a large collection of RIL. The number of significant allele pair imbalances that we detected is surprisingly large and was not expected based on the published evidence.

We have made the top 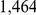 interactions identified with ImAP available as Tables S3 and S4.

## Results

### Overview of the ImAP procedure

**Figure 1:**
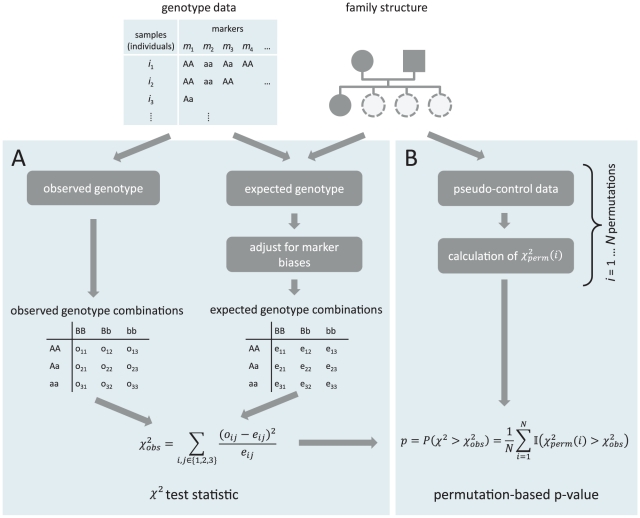
Schematic overview of the test procedure. Panel A shows the calculation of the test statistic, panel B depicts the calculation of the p-values. Family information is used for both parts.

The core step of ImAP consists of a 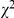-type test comparing the observed frequency of the joint occurrence of a certain diallelic genotype in one locus together with a certain genotype in a second locus with the frequency expected based on the genotypes of the parents under the null hypothesis (i.e. assuming no epistasis, Figure 1). The two loci are required to be distant enough from each other in order not to get false positive results due to local linkage. This results in a score quantifying the deviation of allele pair frequencies from their expected values that is already corrected for inherent population structure. Subsequently, the significance of the scores is assessed with a permutation approach using pseudo-controls that are derived from the genotypes that parents could have transmitted to their offspring. We apply this framework in two steps: First, we only analyze genomic blocks with high local LD using representative markers. In a second step we drill down to individual marker pairs. To further verify our results, we established a simulation procedure that mimics the mating structure of the pedigree under the assumption of independence.

### Mouse genotype data

We applied ImAP to search for potential epistatic interactions using an outbred heterogeneous stock (HS) of mice that was established by crossing eight inbred lines [22]. We are using the genotype data of 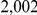 individuals that were genotyped at 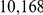 markers. Importantly, the pedigree of these 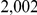 individuals is almost completely known. The HS consists of 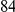 families, some of which are large, while others are only nuclear families. These families were derived from 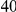 mating pairs of mice from the original stock after more than 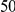 generations of random mating. Genotypes were obtained with the Illumina BeadArray platform achieving call rates of 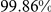, the genotyping accuracy was greater than 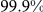 [22].

After removing individuals with more than 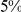 missing data, we were left with 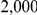 individuals. In addition, we excluded markers with more than 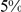 missing values and/or a minor allele frequency (MAF) less than 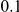. Since we observed a rather poor quality of the genotypes on the X chromosome with relatively few markers passing the quality criteria, we discarded data from this chromosome altogether. The filtering resulted in 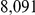 markers used for the subsequent analysis.

We did not have to discard any SNPs due to lack of Hardy-Weinberg equilibrium as is generally done in genome-wide association studies. Instead, ImAP corrects for the disequilibrium (see Methods). In the first run of our analysis, 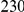 out of 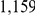 markers had correction factors greater than 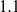 or smaller than 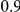. There are several explanations for the deviation from Hardy-Weinberg equilibrium, for example natural selection, genetic drift or segregation distortion [21], [23]. Even though it might not be possible to distinguish the source of disequilibrium, our correction can be applied anyway.

### Testing LD block representatives

When applying ImAP to the HS mouse data, we limited our analysis to markers residing on different chromosomes in order to exclude local LD [18]. An alternative approach would have been to determine local LD first and subsequently apply ImAP to regions outside local LD. As described in the Methods, we first applied ImAP to a reduced set of 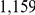 markers, one per LD block.

**Figure 2:**
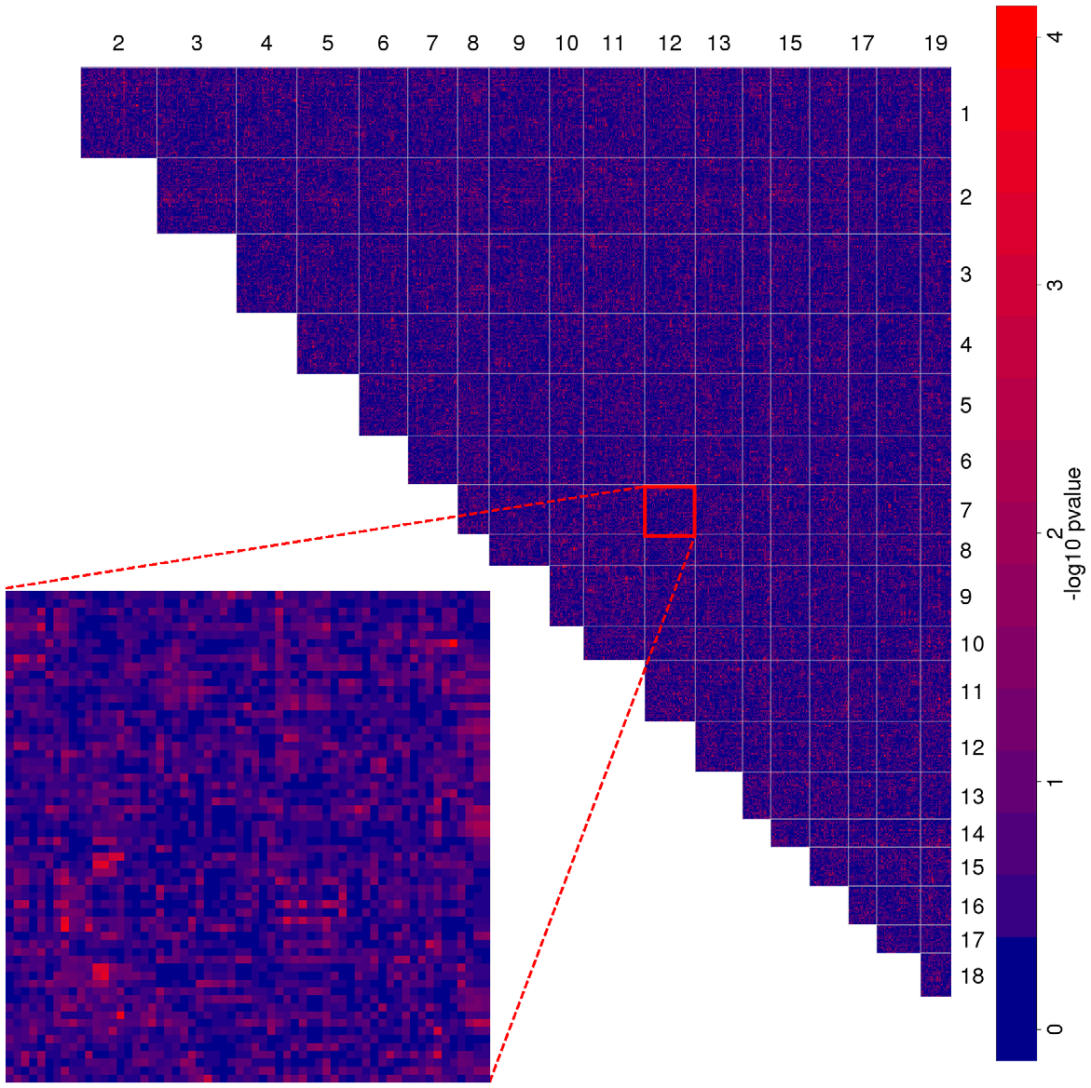
Genome-wide map of allele incompatibilities. The heatmap shows the negative 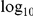 p-values of each LD block combination on different chromosomes. Light red spots show putatively interacting loci. Inset shows an enlargement of chromosome 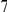 versus chromosome 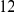.

Figure 2 shows the spatial distribution of the interactions at the level of LD blocks in a genome-wide map. As expected, most block pairs do not interact. At a p-value cutoff of 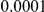 we identify 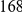 interactions between 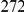 distinct loci (i.e. LD blocks). This p-value corresponds to an FDR of 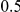 (Benjamini-Hochberg procedure [24]). Although we did not achieve very low FDR values, they were still markedly lower than in five simulated data sets. In two out of these the minimum FDR was above 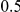.

**Figure 3:**
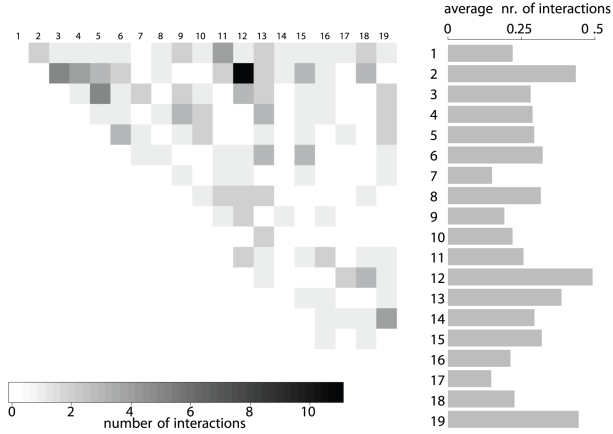
Number of interactions per autosome pair. Results are based on the 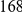 significant LD block pairs involving 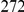 loci. The barplot on the right shows the average number of interactions per LD block for each chromosome. Chromosomes 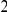, 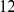, and 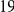 show the highest participation in interactions while the fewest interactions per LD block are on chromosome 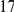.

Most of the loci only interact with one other locus, only 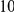 loci participate in more than 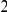 interactions (Figure S5). Not surprisingly, there are more significant interactions between large chromsomes with many measured markers than between small chromosomes (Figure 3). However, we also found markable differences in the relative number of interactions per chromosome. Especially chromosomes 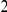, 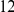 and 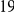 incorporate more loci carrying allelic incompatibilities than other chromosomes. To see whether the number of interactors per chromosome is different from what would be expected by chance, we simulated the 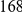 interacting marker pairs 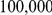 times and compared the distribution of the number of interactors per chromosome to the observed values. At a nominal 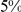 significance level, three chromosomes (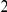, 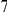, and 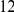) differ from their expected values. At this significance level, we expect less than one chromosome to differ significantly by chance. Hence, there is significant variation of the number of interacting LD blocks between chromosomes.

In order to rule out the possibility of false positive findings due to increased numbers of missing values or small MAF on some markers, we compared the distributions of missing values and MAF between block representatives from significant block pairs to those of non-significant pairs (Figures S3 and S4). There are no significant differences between the proportion of missing values (Wilcoxon rank sum test, p-value 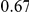). The MAF tends to be even higher in the significant blocks compared to the other blocks. Thus, our results are not biased by missing genotypes or differences in MAF.

**Figure 4:**
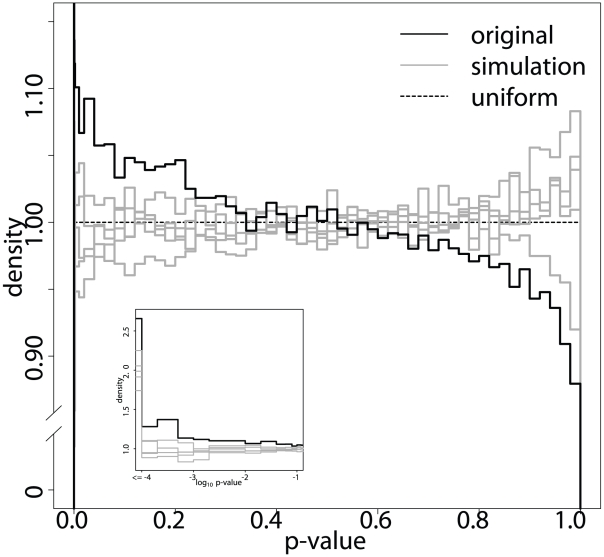
ImAP p-value distribution. Distribution of the p-values of the original data (black) and five simulations under the null hypothesis of no allelic incompatibilities (grey). The y-axis is concentrated on the interesting area of high density. The inset shows a zoom on the small p-values in 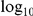 scale.

The histograms in Figure 4 compare the distribution of the p-values that we obtained by applying ImAP to the orignal block representative data with those resulting from five simulations. While the histograms of the simulated data sets resemble those of uniformly distributed p-values under the null hypothesis, the original data show a clear peak in the low p-value range. The simulated pedigrees contain significantly less interactions with low p-values than the real data (one-sided Kolmogorov-Smirnov test 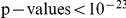). The p-value distribution of the observed genotypes is also significantly different from a uniform distribution (one-sided Kolmogorov-Smirnov test, 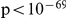). This is not the case for all but one of the simulations (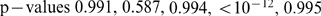). Taken together this confirms that there are more imbalances in allele pair frequencies than expected by chance.

This difference between the real and simulated data can now be quantified to make suggestions about the number of true allelic incompatibilities in the HS mouse population. For example, at 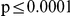 (corresponding to an 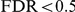) we find between 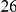 and 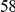 more significant block pairs in the original data compared to the simulations.

As can be seen in the inset of Figure 2, each chromosome pair exhibits only few such interacting pairs that are often surrounded by less significant markers due to local linkage. To further increase the resolution in these interesting regions, we performed fine mapping of all marker pairs in the significant block pairs.

### Fine mapping

For the second step of the analysis we chose all LD blocks that were involved in at least one significant interaction. There might be one or more interacting markers within each LD block and the above analysis does not reveal which markers within a region are involved in the interactions. We repeated the calculation of the test statistics, null distribution and p-values with all markers in those blocks to find the SNP pairs with the highest signal in each significant block pair. This resulted in 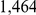 marker pairs with a 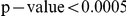 (Tables S3 and S4), since each block pair could contain more than one significant marker pair. Note that the interpretation of the newly calculated p-values has to be done with care since a large number of the tested marker pairs is already assumed to be interacting (they were chosen from interacting LD blocks) and because markers inside LD blocks are highly correlated (i.e. not independent). Therefore, it is difficult to correct for multiple hypothesis testing. However, we can still use the p-values to rank the interactions, i.e. to identify the most likely interacting marker inside each LD block.

### Overlap with allele imbalances in RIL data

Only few allele incompatibilites in mouse have been reported so far [17], [18]. We are not aware of any analysis that quantitatively examines the number of such interactions that can be expected in the whole genome. An overview of the distribution of allele imbalances in RIL is given in [19]. The authors inferred the correlation between locus pairs as a measure for distant LD. The strains used in this study are partly identical to the progenitors of the HS stock. Thus, it is reasonable to assume at least partial overlap of incompatible locus pairs between our study and the RIL data.

We therefore investigated the distant LD of markers that were genotyped in the RIL as well as in the HS mice. We downloaded the genotype data for 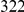 inbred mouse strains (www.genenetwork.org) and recalculated the 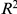 as well as the MAF of the common markers. This allowed us to apply the same quality constraints (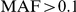) to the RIL data as to the HS genotypes. Moreover, only marker pairs on different chromosomes were considered. After the filtering, 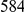 markers constituting 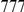 informative pairs were used for the analysis.

Figure S6 compares the overall distribution of distant linkage disequilibrium in the RIL data with that of markers with high ImAP scores. There is a significant difference between the background distribution of 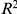 of common marker pairs on different chromosomes and the 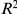 of the top ImAP pairs (one-sided Kolmogorov-Smirnov test, 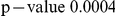). Marker pairs with a significant ImAP score tend to be more in distant LD than other marker pairs. More specifically, 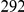 out of the 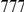 marker pairs have an absolute correlation above 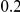. Thus, a significant number of interactions obtained from the HS can independently be confirmed in a different set of mouse populations.

### Functional enrichment

We investigated if the genes mapping to loci that participate in high ranking interactions are enriched for relevant Gene Ontology (GO) categories [25]. ImAP detects interactions between markers, not genes. Thus, in order to perform such analysis we have to assign gene functions to markers. A conservative solution to this problem is to assign to a marker 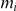 the functions of all genes encoded between the flanking markers 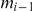 and 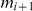. If there actually exists a functional enrichment among genes causing allele incompatibilities this enrichment will be ‘diluted’ due to this procedure. However, since we do not know the causal genes *a priory* there is no other rigorous way of performing such GO enrichment. This strategy also prevents a bias in GO enrichment due to local gene clusters with similar annotation.

We further restricted the enrichment analysis to interacting pairs whose 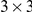 table contained exactly one cell with a zero entry. This corresponds to locus pairs where one allele pair combination was not observed at all in the sample and can thus be assumed to be lethal. We reasoned that genes involved in such an interaction have functions related to organism development. The mapping of genes and their associated GO terms to these markers resulted in 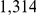 markers having at least one GO term assigned to them. Seventy three of these markers are involved in one of the significant interactions.

The enrichment test was conducted using the topGO algorithm [26]. An advantage of topGO is that it corrects for multiple hypothesis testing, particularly taking into account the nested structure of the GO tree. Since the multiple hypothesis testing correction is inherent in the algorithm, the authors suggest to use the unadjusted p-values as a ranking criterion. We call all terms significant with a 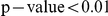 based on the “weighting” algorithm of topGO.

The top ranking GO biological process terms for the original data as well as for an exemplary simulation are shown in the Supporting Material (Tables S1 and S2). We found more significant and more relevant GO terms in the original data compared to the simulation. As expected, many of the significant GO terms are related to developmental processes such as germ cell layer development and development of brain, lung and epithelium. A lot of interesting terms had p-values just above the threshold of 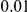 (e.g. stem cell maintenance (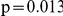), anterior/posterior axis specification (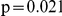) or determination of left/right symmetry (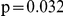)). This analysis shows that markers participating in interactions are enriched for relevant GO categories. One might also expect that pairs of interacting markers share similar functions. However, we did not observe that interacting markers share GO categories more often than expected by chance (data not shown).

### Comparison of interaction profiles

**Figure 5:**
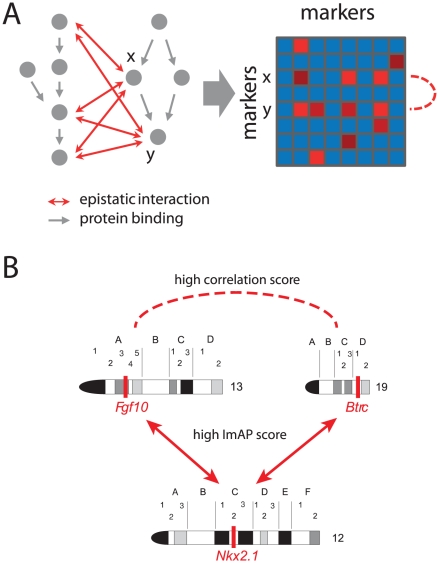
Correlated interaction profiles. (A) Schematic showing relationship between epistatic interactions and molecular pathways. The genes x and y share three allele incompatibilities with genes from a parallel pathway. In the schematic interaction matrix on the right these shared interactions lead to correlated interaction profiles (rows are correlated; dashed line). (B) Example of two loci on chromosomes 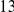 and 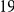 sharing a common interacting locus on chromosome 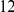. The position of the loci on the chromosomes is indicated by red bars. The putatively causal genes are written below the loci. Arrows indicate interactions with ImAP p-values 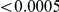, the dashed line indicates a high congruence score (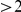).

Epistatic interactions affecting the viability of an organism often bridge parallel pathways [2], [3]. The assumption underlying this between-pathway model is the existence of functional redundancy among pathways. A decrease in functionality of only one of two genes operating in two redundant pathways still allows for regulation of the downstream process through the second alternative pathway. However, if both genes are dysfunctional, both pathways will be disrupted, which may lead to a severe phenotype (i.e. an epistatic interaction between the two genes). Therefore, two genes in the same pathway should share some of their interaction partners, namely those in a functionally similar pathway [27]. Thus, the interaction profiles of genes in the same pathway should be correlated (Figure 5A).

Here, we are interested in markers having a significant number of common interactors. In order to find such groups of markers with similar interaction profiles, we compared the marker interaction profiles from the ImAP analysis using the congruence score [28]. It is calculated as the negative 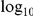 transformed p-value of a hypergeometric test for the number of shared interaction partners. Thus, the score relates the number of interactions shared between two markers to the total number of interactions each single marker participates in [28].

Since here we are analyzing interaction profiles (i.e. all interactions of a given marker rather than single interactions) we chose a less stringent cutoff value for interacting block pairs (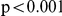). Even though using the more stringent cutoff of 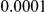 also yielded more correlated interaction pairs in the real data than in the simulations, choosing a higher cutoff increases the difference between real and simulated data. The fraction of block pairs with congruence scores 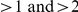 is higher in the original data than in the five simulations (Figure S7). This difference between the proportions is significant in four out of five cases for a significant congruence score (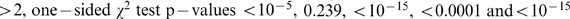). Thus, interaction profiles are more consistent in the real data compared to our simulations.

### Using gene expression data to prioritize candidate genes

An important and nontrivial step in any genetic mapping study is to identify the causal genes encoded in the significant loci. Additional, independent genomic information has been widely used to prioritize genes in a genetic region of interest [29]–[31].

Here, we are using expression data for prioritizing candidate genes at interesting loci. It is likely that several of the allele incompatibilities are caused through functionally relevant changes of gene expression between the minor and major alleles at the two loci [32]. We used expression data from three tissues (lung, liver, hippocampus) measured in a subset of the HS mice (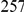, 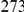 and 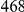 individuals, respectively). For each marker we considered all genes encoded in the region defined by the flanking markers. We then filtered for genes showing significant expression differences between individuals carrying the major versus minor alleles. This analysis was performed independently for each marker using one-way ANOVA with the three possible genotypes as levels. Each genotype had to be observed in at least 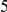 individuals.

Among the 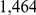 top scoring ImAP pairs, we found 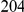, 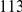 and 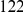 pairs where each locus contained at least one differentially expressed gene (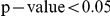) in the hippocampus, liver and lung data sets, respectively. 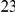 locus pairs were associated with the same differentially expressed genes in all three tissues.

Among the 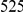 marker pairs with a congruence score greater than 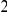 there are 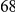, 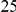 and 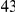 locus pairs containing at least one differentially expressed gene in the hippocampus, lung and liver data, respectively. Figure 5B shows an example of such a marker pair. The putatively causal genes *Fgf10* and *Btrc* showed differential expression (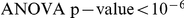) in the hippocampus. The two genes are critically involved in the development of several tissues such as lung, mammary gland, tooth or telencephalon [33]–[37]. This is consistent with the GO terms we found to be enriched among the top scoring ImAP pairs (Table S1). *Btrc* is an inhibitor of Sonic Hedgehog (*Shh*) signaling, which is involved in the development of the lung and the telencephalon [38]. Both, *Fgf10* and *Shh* signaling are involved in development of anatomical structures and are known to influence each other [39].

According to our gene expression analysis, the minor allele of *Fgf10* leads to a reduced expression of this gene while individuals carrying the minor allele of *Btrc* show a higher expression than individuals with the major allele. Since *Btrc* is an inhibitor of *Shh* signaling, this implies that both minor alleles reduce Hedgehog signaling.

The *Btrc* and *Fgf10* loci share 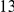 ImAP interactions. One of them involves a locus on chromosome 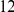 containing, among others, the homeobox transcription factor *Nkx2.1*, which is indispensable for lung and telencephalon development. Depending on the cell type and developmental stage *Nkx2.1* either interacts with the *Fgf10* and *Shh* pathway [38], [40] or it independently acts in parallel [41]. Thus, the reduced activity of Hedgehog signaling in carriers of the minor *Btrc* or *Fgf10* alleles may be rescued by a fully functional *Nkx2.1*. The ImAP analysis suggests that the combination of the minor allele at the *Nkx2.1* locus together with minor alleles at either the *Btrc* or *Fgf10* locus leads to an embryonic lethal phenotype, presumably due the loss of the buffering effect of *Nkx2.1*.

## Discussion

We present a new approach to infer epistatic interactions on a genome-wide scale in family data using sequence information only. The method scans all marker pairs in the genome for deviation from the expected allele pair frequencies resulting in a list of putative pairs featuring an allele incompatibility. Relying on sequence data only is an advantage compared to existing methods for the inference of gene-gene interactions, since the approach can readily be applied to existing SNP data. There is no need for resource- and cost-intensive phenotype measurements.

Regression and 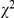 methods have been proposed in the past for the identification of epistatic interactions [1], [7], [9], [10], [42], [43] and the two approaches have been shown to be interconvertible [44]. We chose a 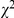-based approach, which makes the fewest assumptions about the underlying genetic model [45]. Which ever way the detection of allele incompatibilities is performed, the key notion is to implement means for accounting for the confounding factors and to remove single-marker effects (e.g. leading to a deviation from Hardy-Weinberg equilibrium). Only after accounting for these confounding factors we got an appreciable number of significant allele incompatibilities.

We identified substantially more interacting loci than expected by chance, which is first evidence that we detect true ‘signal’. Further, we could show that interacting marker pairs are enriched for genes involved in developmental processes and a significant number of interactions could be validated using independent external data. Due to the very large number of pairs tested, finding a large number of interactions with low p-values even in the simulations is expected. However, at low p-values we observed significantly more interactions in the original data than in any of the simulations; e.g. at 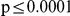 we found at least 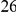 interactions more than in any of the simulations. Considering that so far virtually no allele incompatibilities between mouse strains were reported, this is a surprisingly large number. Suitable statistical tools for the detection of allele incompatibilities at a genomic scale did not exist so far. Hence, this study presents first evidence about the extent of allele incompatibilities in model populations such as the HS. Although the number of interactions we identified might not seem immense, it partly explains the difficulties faced when breeding recombinant inbred lines [19]. For example, during the generation of the Collaborative Cross, a multiparental recombinant inbred strain panel, 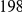 of the 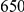 initial lines were lost during the first three to five generations of inbreeding [46]. ImAP helps better understand these issues and it can reveal potential biases in the breeding process that might be introduced due to allele incompatibilities.

Future work should also include haplotype information. Local haplotypes have been inferred for the HS population in terms of probability of inheritance from any of the eight founder strains [47]. I.e. haplotypes are expressed as 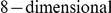 vectors of probabilities. Consideration of these haplotypes would considerably increase the complexity of the analysis (thereby also increasing the number of hypotheses tested), but it might further improve the accuracy.

An epistatic interaction is always defined with respect to a specific phenotype. In this study the phenotype is implicit, hidden. Indeed, looking for allele pairs that are underrepresented in the HS population reveals the genotype of the non-existing individuals. Therefore, the hidden phenotypes should relate to any biological processes affecting the fertilisation, the development or the viability of an individual and thus prevent its appearance in the population. Interestingly, top scoring marker pairs are enriched for genes involved in these expected phenotypes.

It is not immediately obvious how our findings translate to human populations [48], [49]. Although we are working with outbred mice, they were derived from 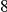 genetically distinct inbred strains. These founder strains differ at at least 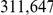 genomic positions (SNPs and structural variations) [50]. It is likely that many of the incompatibilities that we see in the HS developed in the inbred founder strains used for establishing the HS. Even though allele incompatibilities cannot evolve in mixing populations, also human races have been in isolation for more than 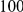 generations [51]–[53]. Hence, it is possible that an appreciable number of incompatibilities exist in the human species. [54] have shown that incompatibilities in yeast can manifest already after relatively few (approximately 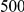) generations. Again, also that finding is not easily transfered to mammals, as the speed of such process will depend on several factors, including recombination- and mutation rates. As the number of family trios that is being fully sequenced increases [55], [56], we expect that our framework will be applicable to human populations within the next years to address these questions.

## Methods

### The ImAP test statistic

The calculation of the test statistic can be divided into several steps which are depicted in Panel A of Figure 1.

1. Let 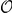 be the set of all individuals for which we have genotype information on the individuals themselves and their parents. This set might differ between markers due to missing values. Hence, for each marker only these trios are taken into account for which there are no missing values in the genotypes of both the parents and the offspring.

2. For each individual in 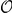, calculate the probability to inherit each genotype based on the genotypes of the parents. This calculation is based on Mendelian laws.

Let 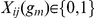 be the genotype indicator of a diploid individual 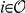 on marker 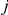. 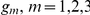, can take one of the three values (AA), (Aa), (aa), where A is the major allele and a the minor allele on marker 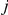. 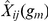 is the corresponding expected genotype probability.

The expected genotype of individual 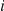 on marker 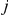 is derived from the genotypes of the parents under the assumption of equal chances of inheriting each of the two possible alleles from each of the parents. The resulting probabilities for all possible parental genotype combinations are shown in Table 1.

**Table 1:** Expected genotype probabilities.

		Offspring		
Parent 1	Parent 2	AA	Aa	aa
**AA**	**AA**	1	0	0
**AA**	**Aa**	0.5	0.5	0
**AA**	**aa**	0	1	0
**Aa**	**Aa**	0.25	0.5	0.25
**Aa**	**aa**	0	0.5	0.5
**aa**	**aa**	0	0	1

Expected genotype probabilities in the offspring for each possible allele combination of the parents.

3. Correct the expected genotypes for possible confounding factors such as segregation distortion. There might be a preference in the inheritance of a certain genotype on one marker in the population which is independent of interaction effects, e.g. if this genotype leads to increased fitness. In order to correct the expected frequencies for allele selection that is independent of other loci we multiply each individual's expected genotype by the ratio of the sample-wide observed and expected frequencies for the corresponding marker (based on all samples):
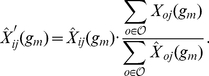
(1)


Normalize the corrected expectation so that the probabilities for each marker sum up to 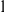:
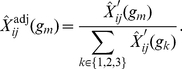
(2)


This guarantees an adjustment of expected allele frequencies in cases where the observed frequency of a marker in the population deviates from the theoretically expected values.

4. Next, the observed and expected number of times each combination of genotypes of two markers appears, can be inferred.

Let 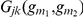 be the observed frequency of the genotype combination 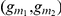 on markers 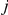 and 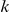, 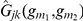 the corresponding expected frequency. They are obtained by summing over all individuals 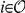:
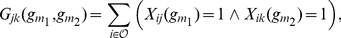
(3)

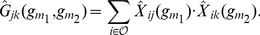
(4)


Using the product of the marginal probabilities of each single marker genotype for calculating the probability of the genotype combination mimics the assumption of no epistatic effects under the null hypothesis. This step results in the 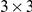 tables in the boxes “observed genotype combination” and “expected genotype combination” in Figure 1.

5. Finally, a 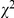-like test statistic can be obtained by first calculating the squared difference of observed and expected frequencies for each genotype combination 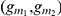 of two markers 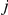 and 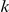 divided by the corresponding expected frequency. The final score for a marker pair is the sum of these values over all nine possible genotype combinations,
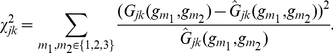
(5)


### Permutation p-values

The significance of the imbalances observed for each marker pair is assessed with a permutation approach based on pseudo-controls. This approach has already been adopted in related problems [57].

The general outline of the procedure is shown in Figure 1B. For each parent-child trio we infer the four genotypes that the child could have inherited at each marker assuming independence. These are then randomly combined to pseudo-offspring genomes in which each of the possible 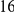 marker pair - genotype combinations could in principle appear. Calculations were done using the R package trio [58].

We use these pseudo genotypes to assess the significance of the test statistics of each marker pair by calculating an empirical marker-specific null distribution based on 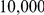 permutations. The permutation p-value is calculated as the fraction of pseudo-control test statistics exceeding the observed score. FDR is obtained with the Benjamini-Hochberg approach [24].

In an earlier version of our analysis pipeline we calculated the p-values based on the 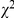 distribution. The degrees of freedom were obtained by using the actual number of genotypes present in the population for each marker pair, 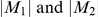. The degrees of freedom are then calculated as 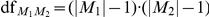.

However, we found that the distribution of these parametric p-values differed conditional on the minor allele frequencies (MAF) of the markers, as shown in Figure S1. The 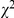 distribution based p-values tend to be too conservative when the MAF is small. The underlying cause is a shift in the distribution of the test statistics depending on the MAF (Figure S2). This phenomenon was greatly reduced when we changed to the permutation based p-value calculation as can be seen in Figure S1.

### Fine mapping of interesting loci

In order to speed up the calculations but still retain an acceptable resolution of loci with potentially interacting genes, we pursued the following strategy.

In a first run of ImAP we split the data into blocks of high linkage disequilibrium (LD). This is again done with the package trio, which provides an algorithm to estimate LD block borders in parent-offspring data. Afterwards, one representative marker is chosen randomly among all markers with a minimum number of missing values in each LD block and the test is applied to all possible combinations of these representatives on different chromosomes. The restriction to markers on different chromosomes is applied to rule out false positive results due to local linkage disequilibrium. Subsequently, we identify all block pairs which were assigned an FDR below 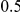 and repeat the analysis using all markers from those blocks. In this way we restrict testing of individual marker pairs to genomic regions that are suggestive for interactions. Finally, we select the highest scoring marker pairs from each locus pair as the ‘interacting pairs’. This two-step approach allows for an accurate mapping of epistatic interactions over the whole genome by simultaneously restricting the number of tests and the computing time to a more reasonable level.

### Pedigree simulation

The pseudo-control data was used to compute p-values. In order to also correct for multiple hypothesis testing and for testing for any other possible biases in our data we simulated the mating process in the mouse population assuming independence of the markers but adhering to the original pedigree structure.

The simulation starts with the first generation of mice for which we have genotype information (F0 generation). Using fastPHASE [59] we infer the haplotypes of these individuals. fastPHASE is based on the notion that haplotypes cluster into locally restricted groups which can be described using a Hidden Markov model. As opposed to other methods, fastPHASE assumes that due to recombination events the group membership changes continuously across the chromosome and not only at the block borders.

Obtaining the haplotypes of the F0 generation allows us to initialize the mating process. For each mother and father of an F1 individual we start with randomly choosing whether they pass on the maternal or the paternal allele of the first marker on a chromosome to the offspring. Then, using either general or sex-specific recombination rates (Supplementary Material in [22]), we sample whether the second marker is inherited from the same chromosome or whether a recombination took place during meiosis. This procedure is continued until a complete chromosome is assembled that is passed on to the offspring. The whole process is repeated until all generations are simulated.

Subsequently, we randomly add 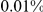 genotyping errors (making sure we do not introduce any Mendelian errors) as well as the same missing values as in the original data.

Since the simulation only accounts for local linkage but not for any other influences on allele frequencies, these data should not contain any true gene-gene interactions. The proportion of false positive findings should be comparable to the original data due to the same error rates and missing values.

## Supporting Information

Figure S1Permutation p-values vs analytical p-values based on the 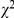 distribution. The colour code shows different MAF of the markers. The smaller the MAF, the more the analytical p-values are conservative.(PDF)

Figure S2Exemplary distributions of the test statistics depending on the MAF of the markers. The scores follow a 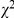 distribution with increasing degrees of freedom for larger MAF.(PDF)

Figure S3 Cumulative distribution functions of the proportion of missing values of representative markers of significant and non-significant LD block pairs.
(PDF)

Figure S4 Cumulative distribution functions of the MAF of representative markers of significant and non-significant LD block pairs.
(PDF)

Figure S5Number of interactions for each of the 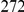 loci involved in the 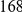 LD block interactions with 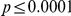. 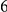, 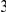 and 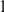 loci have 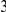, 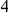 and 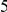 interactors, respectively.(PNG)

Figure S6Cumulative distribution function of the overall distant linkage disequilibrium in the RIL (grey) and RIL marker pairs with ImAP p-value 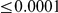 (black).(PDF)

Figure S7Fraction of congruence scores 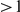 and 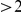 for interaction profiles in original data and five simulations.(PDF)

Table S1 GO enrichment of top ranking marker pairs in the original data. All genes between the flanking markers are considered.
(PDF)

Table S2 GO enrichment of top ranking marker pairs in the simulated data. All genes between the flanking markers are considered.
(PDF)

Table S3Top ranking ImAP interactions. The first two columns contain the IDs of all 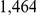 marker pairs with a p-value 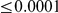 after fine mapping of LD blocks. The third column contains the corresponding 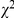 score. The last two columns contain the ImAP p-value and FDR of the marker pair.(XLSX)

Table S4Mapping from marker IDs to Ensembl gene IDs. For each marker 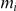 in the first column all genes that are located between its flanking markers 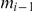 and 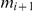 are given in the second column. Gene information is based on Ensembl Build 37 from November 2011.(XLSX)
