# Supplementary figures and images for: Systematic Detection of Epistatic Interactions Based on Allele Pair Frequencies
Source: PLoS Genet. 2012 Feb 9;8(2):e1002463. doi: 10.1371/journal.pgen.1002463 (PMC3276547; doi:10.1371/journal.pgen.1002463)

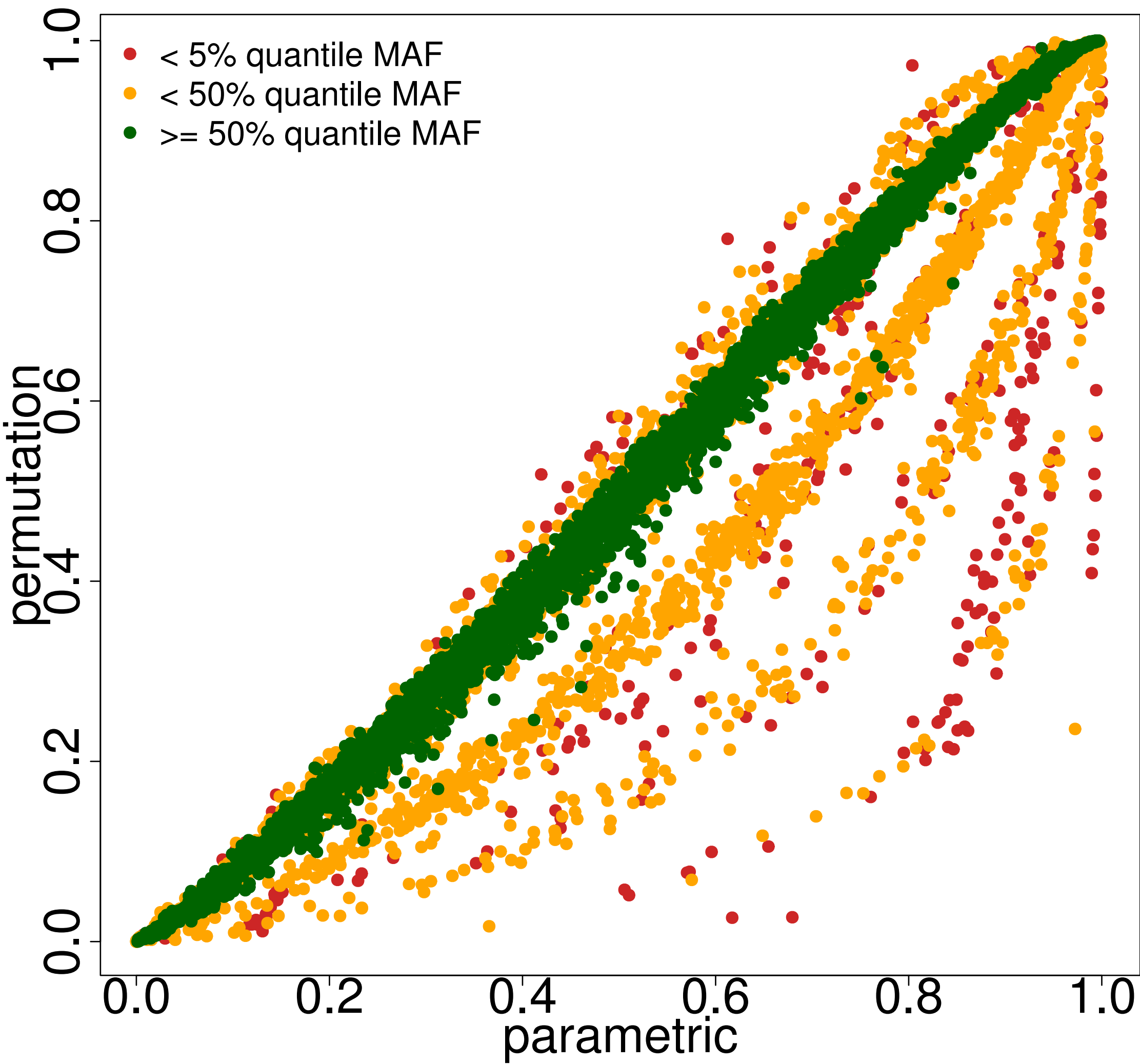

Supplement: Figure S1 — Permutation p-values vs analytical p-values based on the distribution. The colour code shows different MAF of the markers. The smaller the MAF, the more the analytical p-values are conservative. (PDF) [file pgen.1002463.s001.pdf]

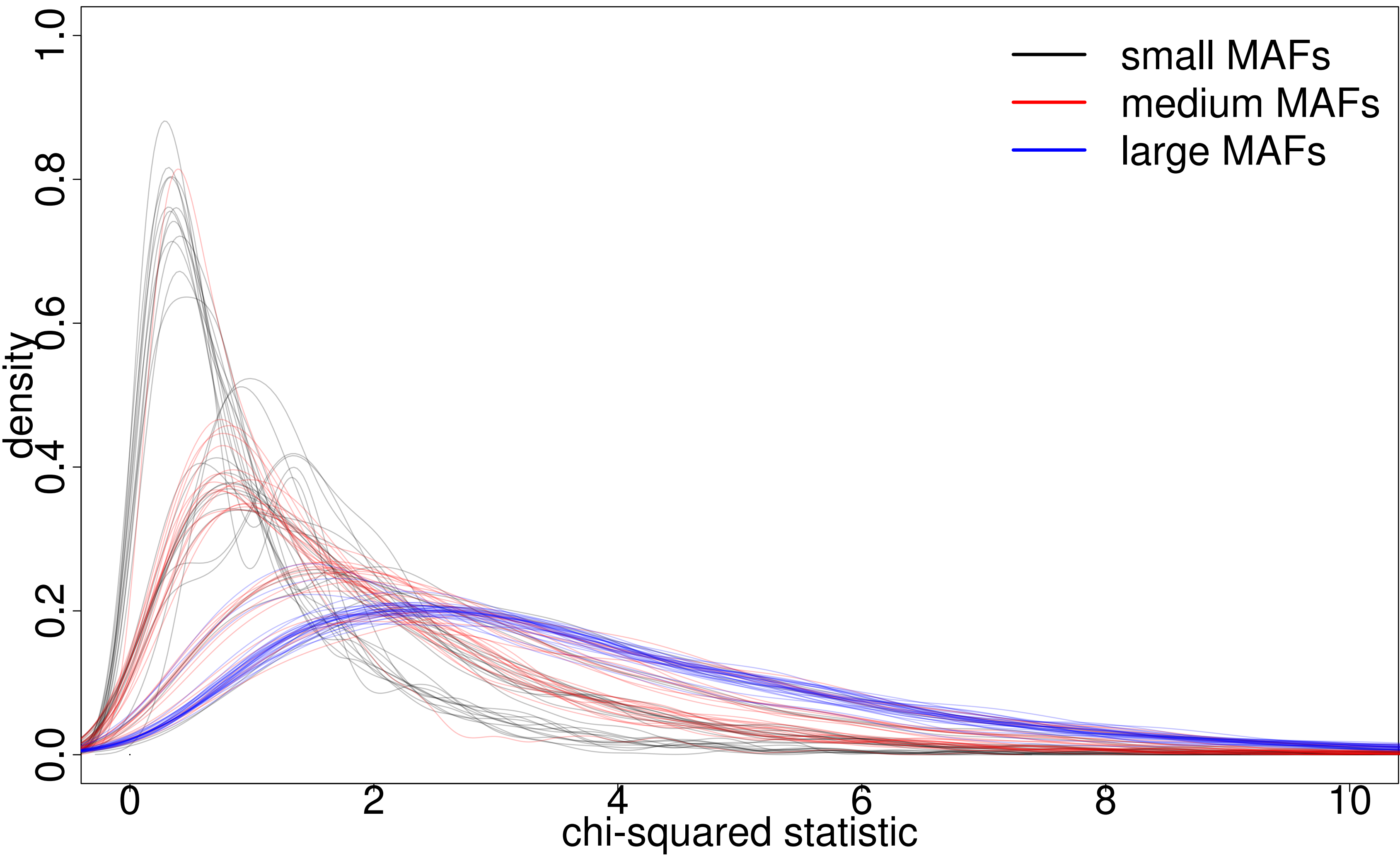

Supplement: Figure S2 — Exemplary distributions of the test statistics depending on the MAF of the markers. The scores follow a distribution with increasing degrees of freedom for larger MAF. (PDF) [file pgen.1002463.s002.pdf]

# proportion of NAs in significant vs non-significant block representatives

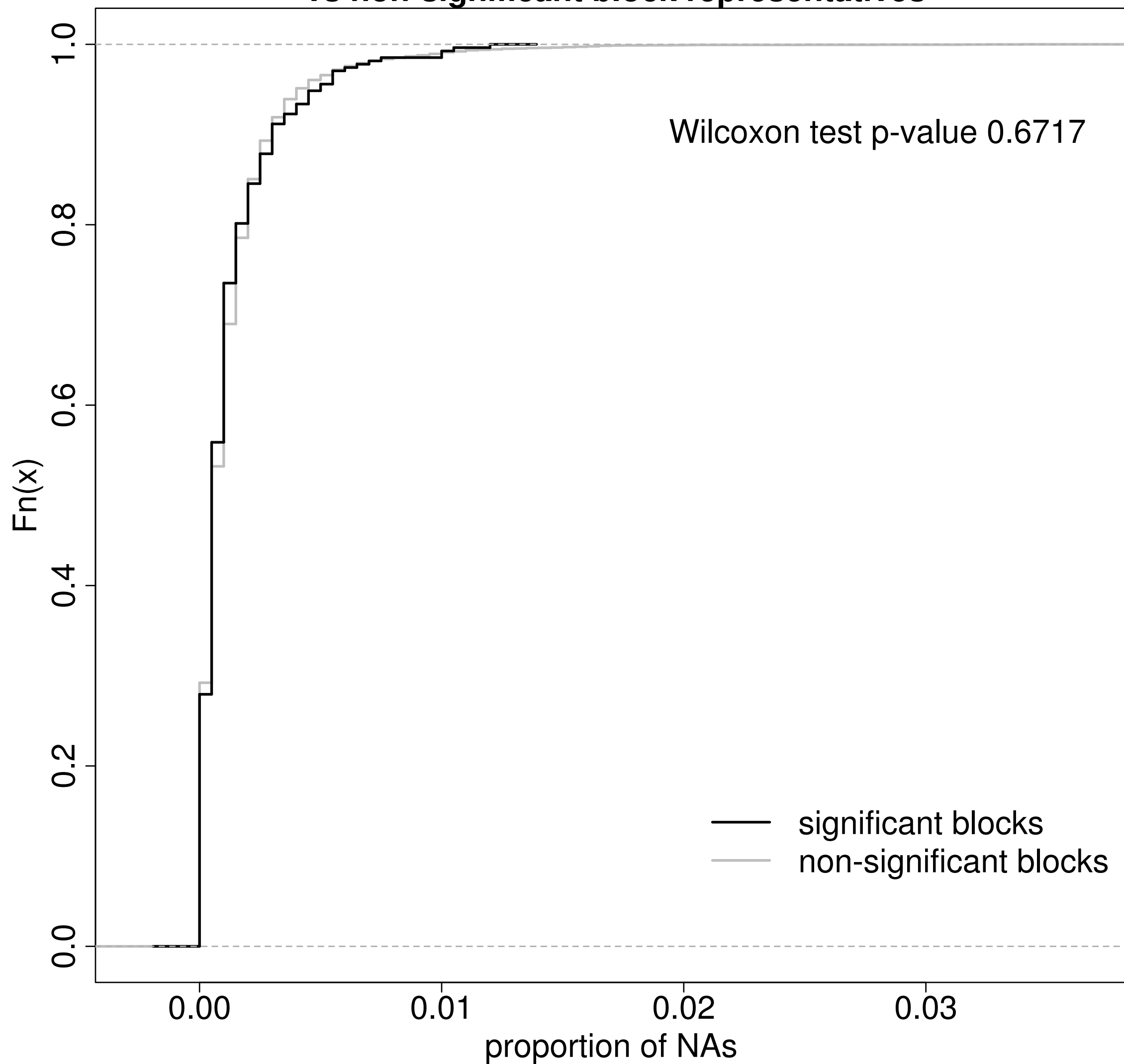

Supplement: Figure S3 — Cumulative distribution functions of the proportion of missing values of representative markers of significant and non-significant LD block pairs. (PDF) [file pgen.1002463.s003.pdf]

**MAF in significant  
vs non-significant block representatives**

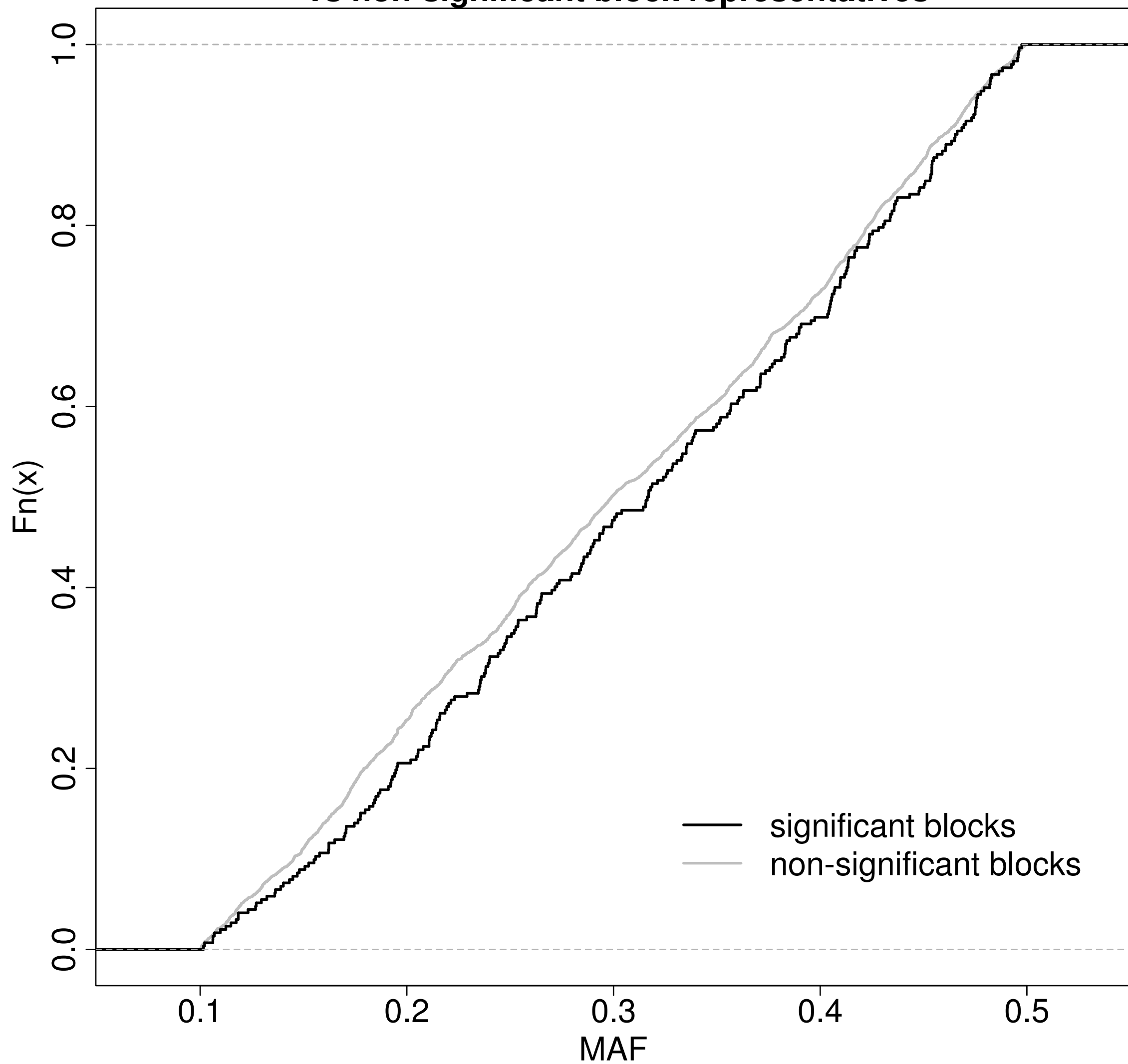

Supplement: Figure S4 — Cumulative distribution functions of the MAF of representative markers of significant and non-significant LD block pairs. (PDF) [file pgen.1002463.s004.pdf]

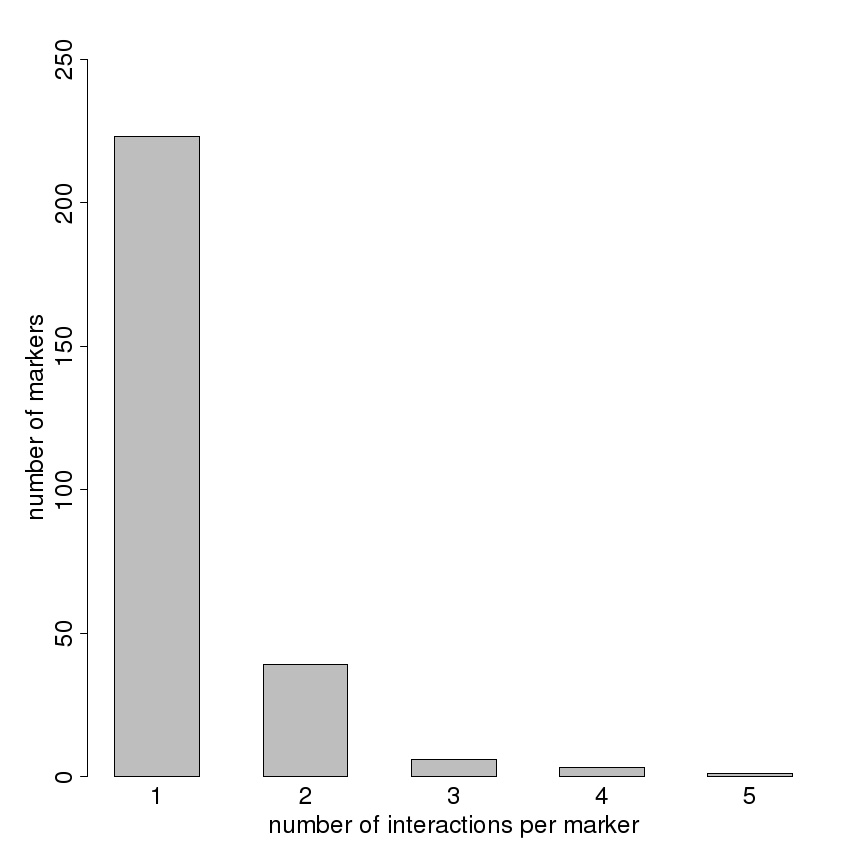

Supplement: Figure S5 — Number of interactions for each of the loci involved in the LD block interactions with . , and loci have , and interactors, respectively. (PNG) [file pgen.1002463.s005.png]

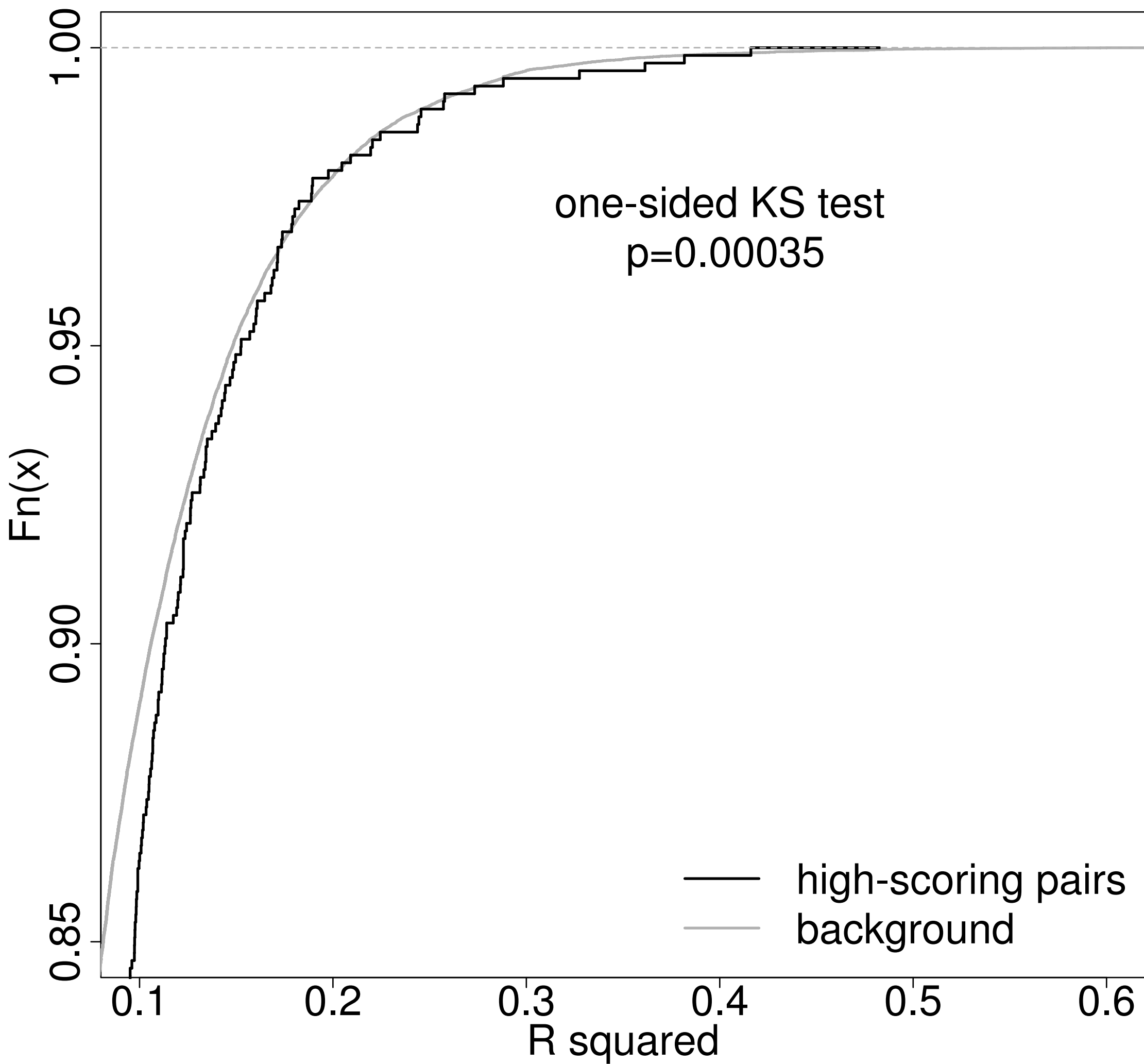

Supplement: Figure S6 — Cumulative distribution function of the overall distant linkage disequilibrium in the RIL (grey) and RIL marker pairs with ImAP p-value (black). (PDF) [file pgen.1002463.s006.pdf]

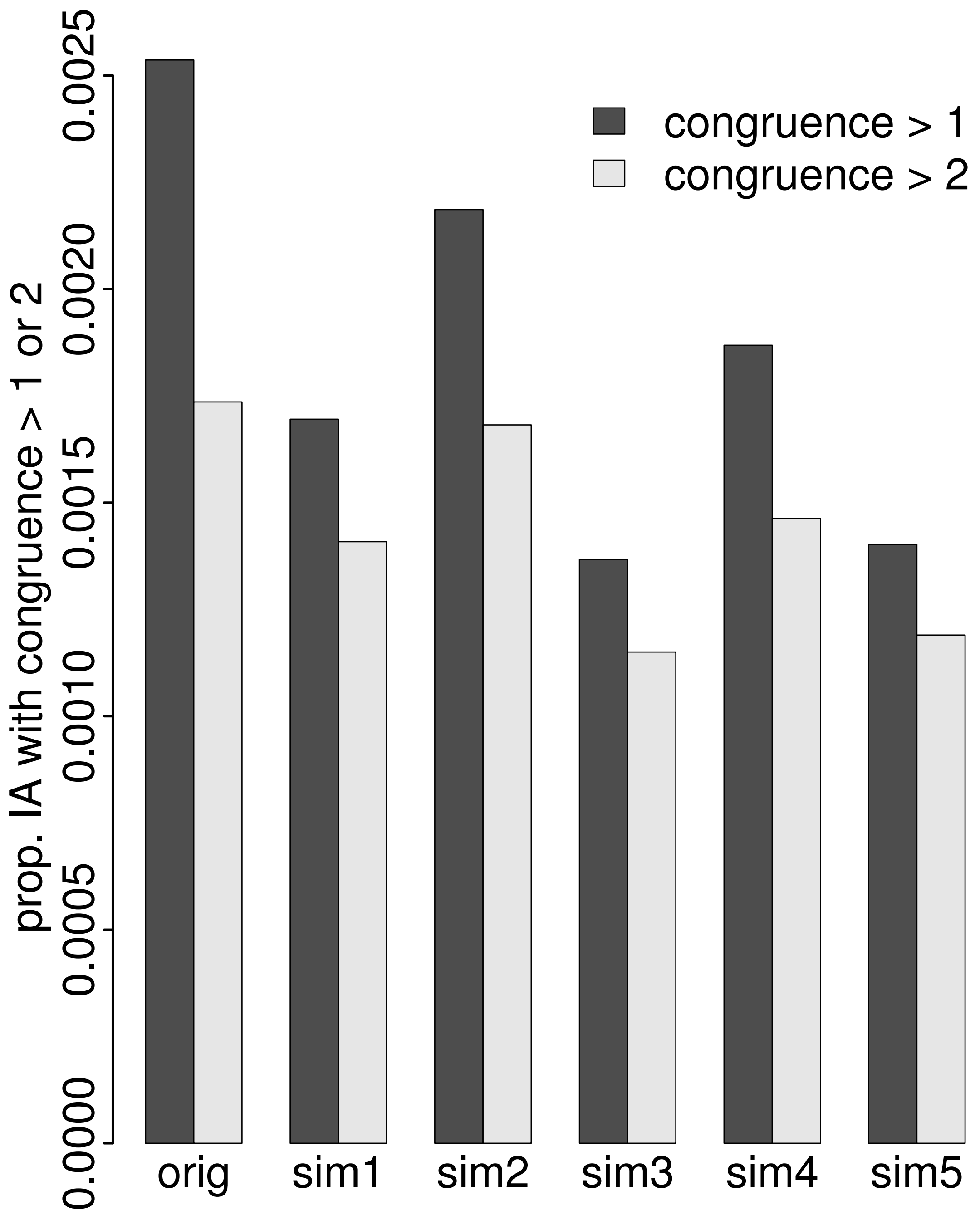

Supplement: Figure S7 — Fraction of congruence scores and for interaction profiles in original data and five simulations. (PDF) [file pgen.1002463.s007.pdf]
