# Supplementary material for: Systematic Detection of Epistatic Interactions Based on Allele Pair Frequencies
Source: PLoS Genet. 2012 Feb 9;8(2):e1002463. doi: 10.1371/journal.pgen.1002463 (PMC3276547; doi:10.1371/journal.pgen.1002463)
Supplement: Table S1 — GO enrichment of top ranking marker pairs in the original data. All genes between the flanking markers are considered. (PDF) [file pgen.1002463.s008.pdf]

**Table S1.**

| GO ID | Term | weighting p-value |
|---|---|---|
| GO:0060592 | mammary gland formation | < 0.00001 |
| GO:0060487 | lung epithelial cell differentiation | < 0.00001 |
| GO:0060441 | branching involved in lung morphogenesis | < 0.00001 |
| GO:0021879 | forebrain neuron differentiation | 0.000046 |
| GO:0032438 | melanosome organization | 0.000046 |
| GO:0030878 | thyroid gland development | 0.000079 |
| GO:0008593 | regulation of Notch signaling pathway | 0.000081 |
| GO:0090130 | tissue migration | 0.000081 |
| GO:0007034 | vacuolar transport | 0.00021 |
| GO:0051345 | positive regulation of hydrolase activity | 0.00022 |
| GO:0060740 | prostate gland epithelium morphogenesis | 0.00031 |
| GO:0032496 | response to lipopolysaccharide | 0.00035 |
| GO:0060788 | ectodermal placode formation | 0.00082 |
| GO:0009880 | embryonic pattern specification | 0.00115 |
| GO:0046638 | positive regulation of alpha-beta T cell differentiation | 0.00124 |
| GO:0022600 | digestive system process | 0.00151 |
| GO:0045931 | positive regulation of mitotic cell cycle | 0.00151 |
| GO:0048839 | inner ear development | 0.0017 |
| GO:0050821 | protein stabilization | 0.0018 |
| GO:0021983 | pituitary gland development | 0.00193 |
| GO:0046579 | positive regulation of Ras protein signaling | 0.00251 |
| GO:0042593 | glucose homeostasis | 0.00306 |
| GO:0060606 | tube closure | 0.00306 |
| GO:0042246 | tissue regeneration | 0.00338 |
| GO:0021761 | limbic system development | 0.00376 |
| GO:0048762 | mesenchymal cell differentiation | 0.00632 |
| GO:0006829 | zinc ion transport | 0.00722 |
| GO:0031128 | developmental induction | 0.00722 |
| GO:0008033 | tRNA processing | 0.00778 |
| GO:0042326 | negative regulation of phosphorylation | 0.00778 |
| GO:0034613 | cellular protein localization | 0.00809 |
| GO:0019882 | antigen processing and presentation | 0.00895 |
| GO:0048730 | epidermis morphogenesis | 0.00895 |
| GO:0006338 | chromatin remodeling | 0.00913 |
| GO:0007050 | cell cycle arrest | 0.00913 |
| GO:0048546 | digestive tract morphogenesis | 0.00913 |
| GO:0007205 | activation of protein kinase C activity by G-protein coupled receptor protein signaling pathway | 0.01 |
| GO:0009268 | response to pH | 0.01 |
| GO:0010948 | negative regulation of cell cycle process | 0.01 |
| GO:0045737 | positive regulation of cyclin-dependent protein kinase activity | 0.01 |
| GO:0048565 | gut development | 0.01063 |
| GO:0001667 | ameboidal cell migration | 0.01229 |
| GO:0019827 | stem cell maintenance | 0.01329 |
| GO:0043616 | keratinocyte proliferation | 0.01329 |

| | | |
|---|---|---|
| GO:0046148 | pigment biosynthetic process | 0.01329 |
| GO:0048146 | positive regulation of fibroblast proliferation | 0.01329 |
| GO:0050654 | chondroitin sulfate proteoglycan metabolism | 0.01329 |
| GO:0051145 | smooth muscle cell differentiation | 0.01329 |
| GO:0090263 | positive regulation of Wnt receptor signaling | 0.01329 |
| GO:0042476 | odontogenesis | 0.01412 |
| GO:0051091 | positive regulation of sequence-specific DNA binding transcription factor activity | 0.01459 |
| GO:0050921 | positive regulation of chemotaxis | 0.0157 |
| GO:0008589 | regulation of smoothened signaling pathway | 0.01712 |
| GO:0018149 | peptide cross-linking | 0.01712 |
| GO:0045666 | positive regulation of neuron differentiation | 0.01831 |
| GO:0009948 | anterior/posterior axis specification | 0.0215 |
| GO:0030512 | negative regulation of transforming growth factor beta receptor signaling pathway | 0.0215 |
| GO:0032312 | regulation of ARF GTPase activity | 0.0215 |
| GO:0042990 | regulation of transcription factor import into nucleus | 0.0215 |
| GO:0048010 | vascular endothelial growth factor receptor signaling pathway | 0.0215 |
| GO:0070374 | positive regulation of ERK1 and ERK2 cascade | 0.0215 |
| GO:0030539 | male genitalia development | 0.02645 |
| GO:0045740 | positive regulation of DNA replication | 0.02874 |
| GO:0031016 | pancreas development | 0.02902 |
| GO:0007368 | determination of left/right symmetry | 0.03194 |
| GO:0031076 | embryonic camera-type eye development | 0.03194 |
| GO:0034976 | response to endoplasmic reticulum stress | 0.03194 |
| GO:0048286 | lung alveolus development | 0.03194 |
| GO:0060560 | developmental growth involved in morphogenesis | 0.03194 |
| GO:0060571 | morphogenesis of an epithelial fold | 0.03194 |
| GO:0060603 | mammary gland duct morphogenesis | 0.03194 |
| GO:0050878 | regulation of body fluid levels | 0.03245 |
| GO:0043627 | response to estrogen stimulus | 0.0348 |
| GO:0001823 | mesonephros development | 0.038 |
| GO:0030318 | melanocyte differentiation | 0.038 |
| GO:0048009 | insulin-like growth factor receptor signaling | 0.038 |
| GO:0048538 | thymus development | 0.038 |
| GO:0050918 | positive chemotaxis | 0.038 |
| GO:0060324 | face development | 0.04459 |
| GO:0060445 | branching involved in salivary gland morphogenesis | 0.04459 |
| GO:0060993 | kidney morphogenesis | 0.04459 |
| GO:0007492 | endoderm development | 0.04677 |
