# Supplementary material for: Systematic Detection of Epistatic Interactions Based on Allele Pair Frequencies
Source: PLoS Genet. 2012 Feb 9;8(2):e1002463. doi: 10.1371/journal.pgen.1002463 (PMC3276547; doi:10.1371/journal.pgen.1002463)
Supplement: Table S2 — GO enrichment of top ranking marker pairs in the simulated data. All genes between the flanking markers are considered. (PDF) [file pgen.1002463.s009.pdf]

Table S2.

GO ID	Term	weighting p-value
GO:0009581	detection of external stimulus	< 0.00001
GO:0010761	fibroblast migration	0.000041
GO:0002474	antigen processing and presentation of peptide antigen via MHC class I	0.000084
GO:0009582	detection of abiotic stimulus	0.00029
GO:0046058	cAMP metabolic process	0.00055
GO:0051320	S phase	0.00102
GO:0048585	negative regulation of response to stimulus	0.00104
GO:0006813	potassium ion transport	0.00193
GO:0007286	spermatid development	0.00298
GO:0006195	purine nucleotide catabolic process	0.00355
GO:0055085	transmembrane transport	0.00388
GO:0009266	response to temperature stimulus	0.00443
GO:0001541	ovarian follicle development	0.00504
GO:0001910	regulation of leukocyte mediated cytotoxicity	0.00512
GO:0006997	nucleus organization	0.00512
GO:0007613	memory	0.00604
GO:0030521	androgen receptor signaling pathway	0.00604
GO:0009207	purine ribonucleoside triphosphate catabolic process	0.00659
GO:0016525	negative regulation of angiogenesis	0.0089
GO:0007018	microtubule-based movement	0.01085
GO:0002707	negative regulation of lymphocyte mediated immunity	0.01253
GO:0030048	actin filament-based movement	0.01253
GO:0045582	positive regulation of T cell differentiation	0.01253
GO:0009416	response to light stimulus	0.01424
GO:0071706	tumor necrosis factor superfamily cytokine production	0.01446
GO:0030198	extracellular matrix organization	0.01471
GO:0006096	glycolysis	0.01526
GO:0030335	positive regulation of cell migration	0.01609
GO:0045333	cellular respiration	0.01789
GO:0002366	leukocyte activation during immune response	0.02345
GO:0010948	negative regulation of cell cycle process	0.02345
GO:0034613	cellular protein localization	0.02435
GO:0006939	smooth muscle contraction	0.02843
GO:0019048	virus-host interaction	0.02858
GO:0055114	oxidation reduction	0.0324
GO:0015833	peptide transport	0.03488
GO:0002064	epithelial cell development	0.03578
GO:0006986	response to unfolded protein	0.03578
GO:0034754	cellular hormone metabolic process	0.04031
GO:0031343	positive regulation of cell killing	0.04196
GO:0042439	ethanolamine and derivative metabolic process	0.04196
GO:0042446	hormone biosynthetic process	0.04394
GO:0048741	skeletal muscle fiber development	0.04394
GO:0034504	protein localization in nucleus	0.04722
